# Effect of uric acid reduction on chronic kidney disease. Systematic review and meta-analysis

**DOI:** 10.3389/fphar.2024.1373258

**Published:** 2024-03-26

**Authors:** Alfredo G. Casanova, Ana I. Morales, Laura Vicente-Vicente, Francisco J. López-Hernández

**Affiliations:** ^1^ Toxicology Unit, Universidad de Salamanca, Salamanca, Spain; ^2^ Instituto de Investigación Biomédica de Salamanca (IBSAL) del Instituto de Ciencias de la Salud de Castilla y León (ICSCYL), Salamanca, Spain; ^3^ Department of Physiology and Pharmacology, Universidad de Salamanca (USAL), Salamanca, Spain; ^4^ Group of Translational Research on Renal and Cardiovascular Diseases (TRECARD), Salamanca, Spain; ^5^ National Network for Kidney Research REDINREN, RICORS2040 RD21/0005/0004-Instituto de Salud Carlos III, Madrid, Spain; ^6^ Group of Biomedical Research on Critical Care (BioCritic), Valladolid, Spain

**Keywords:** uric acid-lowering therapy, chronic kidney disease, protection, prevention, allopurinol

## Abstract

Accumulating evidence suggests that hyperuricemia is a pathological factor in the development and progression of chronic kidney disease. However, the potential benefit afforded by the control of uric acid (UA) is controversial. Individual studies show discrepant results, and most existing meta-analysis, especially those including the larger number of studies, lack a placebo or control group as they aim to compare efficacy between drugs. On these grounds, we performed a me-ta-analysis restricted to studies including the action of any anti-gout therapies referenced to a control or placebo arm. This approach allows for a clearer association between UA reduction and renal effect. Of the twenty-nine papers included, most used allopurinol and febuxostat and, therefore, solid conclusions could only be obtained for these drugs. Both were very effective in reducing UA, but only allopurinol was able to significantly improve glomerular filtration rate (GFR), although not in a dose-dependent manner. These results raised doubts as to whether it is the hypouricemic effect of anti-gout drugs, or a pleiotropic effect, what provides protection of kidney function. Accordingly, in a correlation study that we next performed between UA reduction and GFR improvement, no association was found, which suggests that additional mechanisms may be involved. Of note, most trials show large inter-individual response variability, probably because they included patients with heterogeneous phenotypes and pathological characteristics, including different stages of CKD and comorbidities. This highlights the need to sub classify the effect of UA-lowering therapies according to the pathological scenario, in order to identify those CKD patients that may benefit most from them.

**Systematic Review Registration:** CRD42022306646 https://www.crd.york.ac.uk/prospero/

## 1 Introduction

Chronic kidney disease (CKD) is defined as evidence of abnormalities in renal structure or function for at least 3 months, with implications for health (Stevens, 2013) including increased bone and cardiovascular morbidity, and significantly reduced life expectancy and quality of life (Ortiz, 2022). Patients in terminal stages (i.e., a 0.13% of the whole population) become dependent on costly replacement therapy in the form of dialysis or renal transplant (Ortiz, 2022). Due to its high prevalence and the absence of an effective treatment, CKD is one of the leading causes of mortality worldwide (Kovesdy, 2022). It is estimated that 10% of the adult population suffers from some degree of CKD, and that by 2100 CKD will be the second leading cause of death from disease (Ortiz, 2022). These data show the need to develop strategies to reduce these numbers, and measures for the prophylactic mitigation of its causes. Several factors have been associated with the risk of developing CKD. Non-modifiable factors include age, sex, race or family history, while others can be averted, treated pharmacologically or reduced, such as obesity, hypertension, diabetes and the use of tobacco or analgesics (Kazancioğlu, 2013). In the last decades, hyperuricemia has been proposed as a potential risk factor for CKD (Feig, 2020).

Hyperuricemia, an excess of uric acid (UA) in the blood, is a common cause of gout (Brook et al., 2010). In 1940, it was observed that a high number of patients with gout also suffered from kidney disorders (Coombs et al., 1940). A later study indicated that 90% of autopsied patients with gout also had kidney damage (glomerulosclerosis, tubulointerstitial fibrosis, and arteriosclerosis) (Talbott and Terplan, 1960). It was suggested that kidney damage was caused by the deposit of urate crystals found in the tubules and the interstitium in these patients. Subsequent studies with animal models confirmed that urate crystals directly cause tubular damage, in part mediated by oxidative stress (Sánchez-Lozada et al., 2002). However, additional pathological factors related to hyperuricemia could exist because urate deposits have also been detected in gouty patients with no evidence of renal damage (Yü and Berger, 1982). Furthermore, a preclinical study determined that mild and transient hyperuricemia, in the absence of urate deposits, also aggravated and accelerated CKD (Mazzali et al., 2001). Other proposed mechanisms of kidney damage caused by hyperuricemia include mitochondrial dysfunction, renin-angiotensin system overactivation, and endothelial dysfunction produced by a reduction of nitric oxide and excessive release of vasoconstrictors (e.g., endothelin and thromboxane) (Mazzali et al., 2001; Mallat et al., 2016; Bonino et al., 2020).

If hyperuricemia plays a significant role in CKD, reducing its levels should have a beneficial effect on renal function (i.e., slowing down or reverting disease progression). In this regard, controversial evidence exists. While numerous clinical studies support a renoprotective effect of hypouricemic therapy [reviewed in (Richette et al., 2018)], others did not find a positive association [reviewed in (Leoncini et al., 2022)]. Meta-analyses that have been performed to date (Bose et al., 2014; 2014; Chen et al., 2020; Sun et al., 2020; Liu et al., 2021; Tsukamoto et al., 2021; Gonçalves et al., 2022) included trials in which the comparator was placebo, usual care, or an alternative medicine, in a mixed manner. Almost invariably, existing meta-analyses only ascribe protection to those treatments that improve renal function and, thus, may underestimate their efficacy. A strict comparison *versus* placebo/control is necessary to discern whether anti gout treatments improve renal function or merely slow down its progressive decay. Accordingly, with the objective of studying the effect of hyperuricemia-lowering strategy on renal function in CKD, we meta-analyzed only those studies containing a placebo/control arm and carried out a correlation study between hypouricemic efficacy and renal protection.

## 2 Methods

The protocol of this systematic review is registered in PROSPERO with the code CRD42022306646 (25/02/2022). The entire procedure described below was carried out in accordance with the Preferred Reporting Items for Systematic Reviews and Meta-Analyses (PRISMA) guidelines.

### 2.1 Systematic study mining

A bibliographic search of articles published up to September 2023 in MEDLINE and the Web of Science databases was carried out. MeSH terms and keywords were used in order to maximize article mining. In the PUBMED platform, the MeSH terms used were “Chronic Renal Insufficiency” [Mesh]) and “Gout Suppressors” [Pharmacological Action]. In both platforms, the terms were used independently as follows “((“Kidney Failure, Chronic”) OR (“Chronic Kidney Failure”) OR (“Chronic Kidney Injury”) OR (Renal Failure, Chronic) OR (Chronic Renal Injury) OR (chronic renal disease) OR (CKD) OR (CKF)) AND ((“Gout Suppressants”) OR (Allopurinol) OR (Benzbromarone) OR (benziodarone) OR (Colchicine) OR (Febuxostat) OR (halofenate) OR (Probenecid) OR (sulfinpyrazone) OR (tricrynafen) OR (zoxazolamine) OR (pegloticase) OR (rasburicase) OR (losartan))”. In both cases, the human filter was added to select only clinical trials. Subsequently, an additional search was carried out introducing each drug individually along with the terms specified for kidney damage, which further enhanced article identification.

### 2.2 Inclusion and exclusion criteria

First, two members of the research team (A.G.C. and L.V.-V.) independently identified those studies that met at least one of the following exclusion criteria: 1) reviews, protocols, case-reports, congress abstracts, editor letters or comments; 2) pre-clinical studies; 3) only abstract available; 4) unrelated content; or 5) language other than English, Spanish, Italian, French or Portuguese. Among the remaining studies, only those that met all the following inclusion criteria were definitively selected: 1) Randomized studies in which urate-lowering therapy is administered in patients with CKD; 2) Studies that evaluate renal function by estimated Glomerular Filtration Rate (eGFR), serum creatinine (sCr), albuminuria or proteinuria [reporting the mean and a measure of dispersion that allow calculation of the standard deviation (SD)]; 3) Studies that present baseline and follow-up data; and 4) Studies that include a control or placebo group. After comparing the list of articles selected by both researchers, a third member of the team (A.I.M.) was designated to resolve potential discrepancies.

### 2.3 Data extraction

The following data were extracted from each selected article: study reference (first author and year of publication), design, location, patient recruitment dates, type of population, number of patients in the treated group and in the placebo/control group, evaluated treatment (drug, dose and duration of treatment). Clinical study design quality was calculated according to the Jadad scale (Jadad et al., 1996) (studies with a score of five were considered rigorous, scores between three and five were considered good quality, and scores below three were considered poor quality (and were eliminated). Additionally, the mean SD values of the parameters serum uric acid (sUA), eGFR, sCr, albuminuria and/or proteinuria were registered (or calculated from the standard error of the mean or the confidence interval). When verifying that only two studies evaluated albuminuria, it was decided to dispense with this biomarker. From these numerical data, the mean increase in each biomarker (BM_Δ_) was calculated in the treated and the control/placebo groups with the formula: BM_Δ_ = BM_F_ − BM_B_, where BM_F_ is the mean value of the biomarker at the end of the nephroprotective treatment, and BM_B_ is the mean baseline value of the biomarker. The standard deviation resulting from this difference, s_Δ_, was also calculated as the accumulation of errors: s_Δ_ = $\sqrt{s_{F}^{2}+s_{B}^{2}}$ , where $s_{F}$ is the SD value of the biomarker at the end of the nephroprotective treatment, and $s_{B}$ is the SD value of the biomarker at baseline. Since most of the included studies evaluated the drugs allopurinol and febuxostat, these analyzes were performed independently for each of these treatments and for an additional group of drugs called ‘Others'.

### 2.4 Meta-analysis

Heterogeneity between studies was assessed with the Cochran’s Q test under the null hypothesis of homogeneity (*p* < 0.05 indicated heterogeneity) and the I^2^ index (I^2^ > 50% indicated high heterogeneity). After this, the fixed-effects model (for homogeneous studies) or the random-effects model (for heterogeneous studies) was selected to meta-analyze the data. The Hedges’ g value and its 95% confidence interval were calculated for each study and each renal function biomarker with the following formula:

$$g=\frac{{BM}_{∆T}-{BM}_{∆C/P}}{sp}$$

where:

$$sp=\sqrt{\frac{\left(n_{T}-1\right)s_{∆T}^{2}+\left(n_{C/P}-1\right)s_{∆C/P}^{2}}{\left(n_{T}-1\right)+\left(n_{C/P}-1\right)}}$$

where *BM*_Δ*T*_ and *BM*_Δ*C/P*_ are the biomarker increases in the treatment and in the control/placebo groups, respectively; $s_{∆T}^{2}$ and $s_{∆C/P}^{2}$ are the standard deviations of the treatment and the control/placebo groups, respectively; and $n_{T}$ and $n_{C/P}$ correspond to the sizes of the treatment and control/placebo groups, respectively. Forest plots were constructed in which the g parameters of the different included studies were compared.

Finally, funnel plots in which the Hedges’ g of each study was represented *versus* its standard error were constructed to evaluate potential publication bias. In addition, the asymmetry tests of Begg and Mazumdar (Begg and Mazumdar, 1994) and Egger et al. (Egger et al., 1997) were applied. All the analyses described in this section were carried out with the *Meta-Essentials* set of workbooks (Suurmond et al., 2017).

### 2.5 Correlation study

In order to study the relationship between the ability of the tested treatments to reduce sUA levels and to improve renal function, a Pearson correlation test was performed (for normal data, which was previously verified with the Saphiro-Wilk test). Only the treated groups of those studies that quantified both evolution in sAU and eGFR from the start to the end of treatment were included. *p*-values lower than 0.05 were considered statistically significant. This analysis was performed with the IBM SPSS Statistics 20.0 software (International Business Machines, Armonk, NY, United States).

## 3 Results

### 3.1 Description of included studies

**FIGURE 1:**
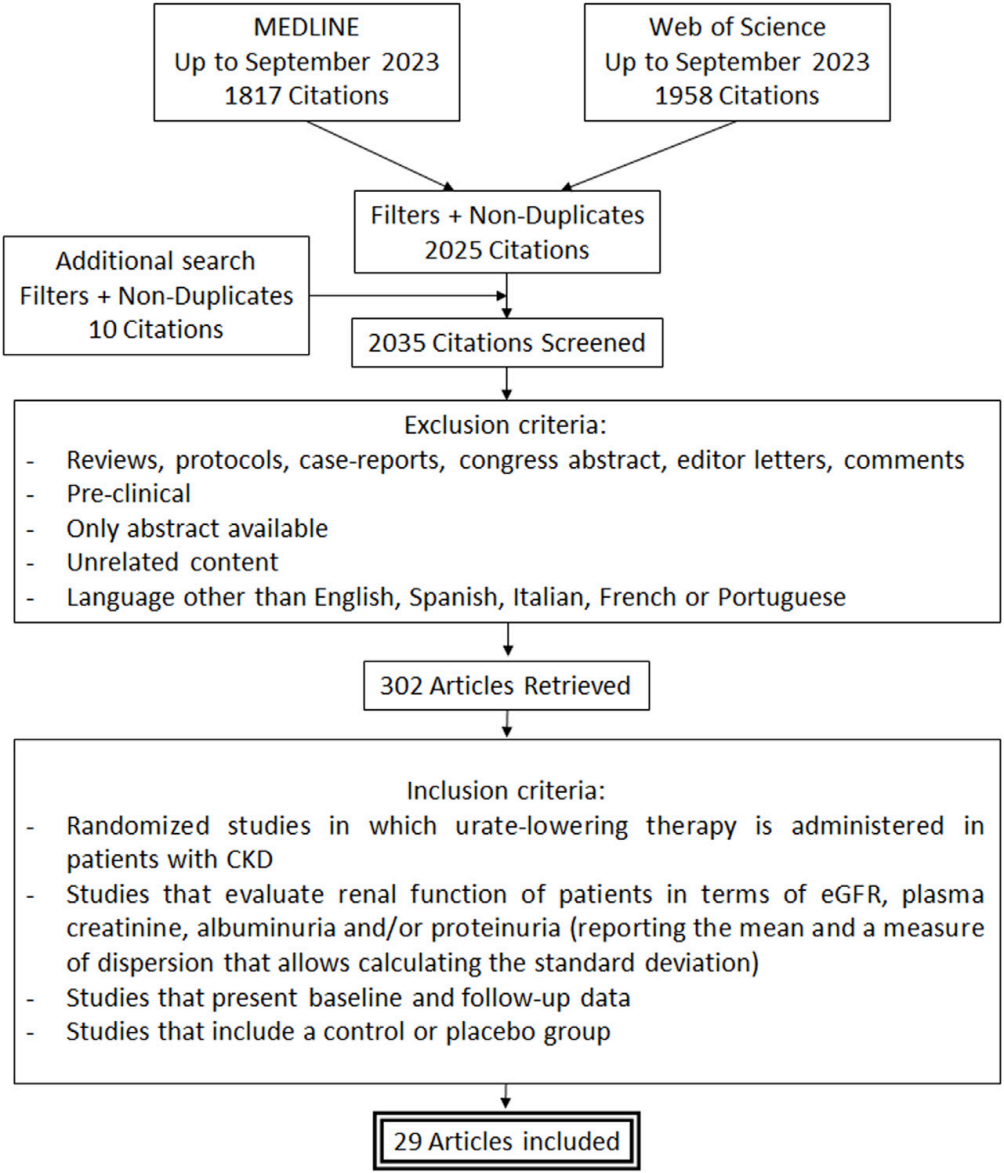
Flowchart of the search process.

A flowchart of the search process followed for the selection of the 29 clinical studies finally included is presented in Figure 1 (Katholi et al., 1998; Siu et al., 2006; Kanbay et al., 2007; Malaguarnera et al., 2009; Nouri-Majalan, 2009; Zhu et al., 2009; Goicoechea et al., 2010; 2015; Momeni et al., 2010; Kao et al., 2011; Shi et al., 2012; Sezer et al., 2014; Bayram et al., 2015; Sircar et al., 2015; Golmohammadi et al., 2017; Krishnamurthy et al., 2017; Ghane Sharbaf and Assadi, 2018; Kimura et al., 2018; Wada et al., 2018; Johnson et al., 2019; Lee and Lee, 2019; Badve et al., 2020; Doria et al., 2020; Perrenoud et al., 2020; Wen et al., 2020; Jeyaruban et al., 2021; Stack et al., 2021; Nata et al., 2023; Yang et al., 2023).

The descriptive data extracted from the clinical studies included in this meta-analysis are shown in Table 1. Total patients add up to 4,471 (44.1% receiving an UA-lowering therapy). The drug predominantly used is allopurinol followed by febuxostat.

**TABLE 1:** Characteristics of the clinical studies included in this meta-analysis. CKD, chronic kidney disease; eGFR, estimated glomerular filtration rate; i.v., intravenous; n.a., not applicable; n.d., not described; p.o., per os (orally); Prot, proteinuria; SCr, serum creatinine concentration; sUA, serum uric acid concentration.

Study identification	Design	Location	Duration of recruitment	Population	Patients initially included (Treatment/Control or placebo group)	Tested treatment	sUA	Renal function biomarkers	Jadad score
Badve et al. (2020)	Prospective, randomized, double-blind trial	Australia and New Zealand	March 2014–December 2016	Adults with CKD stage 3–4 and no history of gout	182/181	Allopurinol 100 mg/day (the first 12 weeks) and up to 300 mg/day (until the end of the study) p.o. for 96 weeks	Yes	eGFR	5
Bayram et al. (2015)	Prospective, randomized, open-label trial	Turkey	January 2012–November 2012	Adults with CKD at stage 2–4, having sUA levels over 5.5 mg/dL	30/30	Allopurinol 300 mg/day p.o. for 12 weeks	Yes	eGFR, Prot	4
Doria et al. (2020)	Prospective, double-blind, multicenter, randomized, placebo-controlled clinical trial	United States of America	2013–2016	Adults with diabetes mellitus 1, eGFR between 40 and 99.9 mL/min/1.73 m2 and sUA ≥4.5 mg/dL	267/263	Allopurinol 100 mg/day for 4 weeks and 300–400 mg/day p.o. until 156 weeks	Yes	eGFR	5
Ghane Sharbaf and Assadi (2018)	Prospective, randomized, open-label trial	Iran	January 2015–July 2017	Children (3–15 years) with CKD stages 1–3 and elevated sUA (>5.5 mg/dL)	38/32	Allopurinol 10 mg/kg/day (maximum daily dose 300 mg) p.o. for 16 weeks	Yes	eGFR	3
Goicoechea et al. (2010)	Prospective, randomized open-label trial	Spain	January 2007–May 2007	Adults with eGFR <60 mL/min but stable renal function, no hospitalized, nor cardiovascular events	57/56	Allopurinol 100 mg/day for 96 weeks	Yes	eGFR	5
Goicoechea et al. (2015)	Prospective, randomized open-label trial	Spain	January 2007–May 2007	Adults with eGFR <60 mL/min but stable renal function, no hospitalized, nor cardiovascular events	57/56	Allopurinol 100 mg/day for 240 weeks	Yes	eGFR	5
Golmohammadi et al. (2017)	Prospective, randomized, blind trial	Iran	January 2014–December 2015	Adults with eGFR between 15 and 60 mL/min/1.73 m2	96/100	Allopurinol 100 mg/day p.o. for 48 weeks	Yes	eGFR, SCr	4
Jeyaruban et al. (2021)	Retrospective cohort study	Australia	May 2011–August 2017	Adults who had been commenced kidney replacement therapy prior to May 2011	207/916	Allopurinol, prescribed dose as anti-gout treatment	No	eGFR	n.a
Johnson et al. (2019)	Prospective, multicentre, randomized, double-blind trial	United States of America	May 2006–October 2007	Adults with chronic gout	169/43	Pegloticase 8 mg every 2 weeks or every 4 weeks p.o. for 24 weeks	No	eGFR	5
Kanbay et al. (2007)	Prospective, randomized open-label trial	Turkey	n.d	Adults with eGFR >60 mL/min with sUA levels >7.0 mg/dL	48/21	Allopurinol 300 mg/day for 12 weeks	Yes	eGFR, SCr, Prot	3
Kao et al. (2011)	Prospective, randomized, double-blind, parallel-group trial	United Kingdom	January 2008–December 2008	Adults who were diagnosed left ventricular hypertrophy and to have CKD stage 3	32/35	Allopurinol 100–300 mg/day p.o. for 36 weeks	Yes	eGFR, Prot	5
Katholi et al. (1998)	Prospective, randomized, double-blind, trial	United States of America	n.d	Adults with Crpl between 1.4 and 2 mg/dL	22/17	Allopurinol 4 mg/kg/day p.o. (starting 24 h before administration of contrast media) for 3 days	No	eGFR	5
Kimura et al. (2018)	Prospective, multicenter, randomized, double-blind, parallel-group, placebo-controlled trial	Japan	November 2012–December 2013	Adults who have hyperuricemia without gouty arthritis, who present CKD stage 3, and whose sUA concentration is 7.1–10.0 mg/dL	200/200	Febuxostat 10 mg/day p.o. at weeks 1–4 after study initiation, increased to 20 mg/day at weeks 5–8 and elevated to 40 mg/day at week 9 until week 108	No	eGFR	5
Krishnamurthy et al. (2017)	Retrospective cohort study	United States of America	October 2000–November 2006	Adults who have sUA greater than 7 mg/dL	50/50	Allopurinol 221 mg/day (average dose) for the time prescribed as anti-gout therapy	Yes	eGFR, SCr	n.a
Lee and Lee (2019)	Retrospective cohort study	South Korea	June 2005–April 2018	Adults with CKD stage 3 and hyperuricemia	Febuxostat 30/Allopurinol 40/No treatment 71	Febuxostat 20, 40 or 80 mg/day or allopurinol 100, 200 or 300 mg/day for 224 weeks (average)	Yes	eGFR	n.a
Malaguarnera et al. (2009)	Prospective, randomized, placebo-controlled trial	Italy	January 2004–November 2005	Adults with hyperuricaemia (>7 mg/dL)	20/18	Rasburicase 4.5 mg in 10 cc physiological solution single dose i.v. infusion	Yes	eGFR, SCr	5
Momeni et al. (2010)	Prospective, randomized, double-blind, controlled trial	Iran	August 2006–May 2008	Adults with proteinuria greater than 500 mg/day bilateral normal size kidney on ultrasonography (9 cm–12 cm), existence of diabetic retinopathy, and absence of systemic diseases or other causes of proteinuria	20/20	Allopurinol 100 mg/day p.o. for 16 weeks	Yes	SCr, Prot	5
Nata et al. (2023)	Prospective, randomized trial	Thailand	February 2018 –February 2019	Adults with stages 3 or 4 CKD with asymptomatic hyperuricemia	40/37	Febuxostat 40 mg/day for 8 weeks	yes	eGFR	3
Nouri-Majalan (2009)	Prospective, randomized trial	Iran	March 2006–March 2008	Adults with acute renal failure, GFR <60 mL/min and undergoing cardiac coronary artery bypass graft	30/30	100 units vitamin E 4 times per day and allopurinol 200 mg/daily for 3–5 days prior to elective surgery	No	eGFR, SCr	3
Perrenoud et al. (2020)	Prospective, double-blind randomized placebo-controlled study	United States of America	October 2010–September 2016	Adults with stage 3 CKD and asymptomatic hyperuricemia	33/36	Allopurinol 300 mg/day p.o. for 12 weeks	No	eGFR	5
Sezer et al. (2014)	Cross-sectional study	Turkey	n.d	Adults with stage 3–4 CKD	39/47	Allopurinol mean dose 1.5 ± 0.8 mg/kg/day for 48 weeks	Yes	eGFR	4
Shi et al. (2012)	Retrospective cohort study	China	January 1993–December 2006	Adults with chronic glomerulone-phritis with IgA immunoglobulin	21/19	Allopurinol 100–300 mg/day for 24 weeks	Yes	eGFR, Prot	5
Sircar et al. (2015)	Prospective, double-blind, randomized, placebo-controlled trial	India	February 2012–January 2013	Adults with eGFRs of 15–60 mL/min/1.73 m2 and serum uric acid levels ≥7 mg/dL	45/48	Febuxostat 40 mg/day p.o. for 24 weeks	Yes	eGFR	5
Siu et al. (2006)	Prospective, randomized, controlled trial	China	April 2003–April 2004	Adults with renal disease (daily proteinuria greater than 0.5 g and/or an elevated SCr level >1.35 mg/dL (120 mol/L) at baseline	25/26	Allopurinol 100 or 200 mg/day for 48 weeks	Yes	SCr, Prot	3
Stack et al. (2021)	Prospective, multicenter, randomized, double-blind, parallel group, placebo-controlled trial	United States of America	May 2017–August 2018	Adults with hyperuricemia, albuminuria, and type 2 diabetes mellitus	32/28	Febuxostat 80 mg/day p.o. + Verinurad 9 mg/day p.o. for 24 weeks	Yes	eGFR, SCr	5
Wada et al. (2018)	Prospective, multicenter, randomized, double- blind, placebo-controlled, parallel-group study	Japan	n.d	Adults with diabetic kidney disease, diagnosed with gout or hyperuricemia	43/22	Topiroxostat 40 mg/day for 4 weeks followed by stepwise increase of the dose 160 mg/day 28 weeks	Yes	eGFR	5
Wen et al. (2020)	Prospective, randomized, controlled trial	China	n.d	Adults with CKD stage 3 and diabetic nephropathy complicated by high serum uric acid (360 μmol/L)	18/20	Febuxostat 20 mg/day p.o. for 4 weeks	Yes	eGFR, SCr, Prot	3
Yang et al. (2023)	Prospective randomized controlled trial	China	August 2026–May 2019	Adults with CKD stage 3–4	47/45	Febuxostat 20 m/day for 2 weeks and 40–80 mg/day for almost 48 weeks	yes	eGFR	3
Zhu et al. (2009)	Prospective, open, randomized, case-control study	China	February 2005–March 2007	Adults with kidney transplant and that had stable graft function as evidenced by SCr concentration less than 176.8 mol/L and Hb concentration greater than 110 g/L	34/32	Losartan 50 mg/day for 24 weeks	Yes	eGFR, SCr	3

### 3.2 Results of the meta-analysis

#### 3.2.1 Hypouricemic efficacy

**FIGURE 2:**
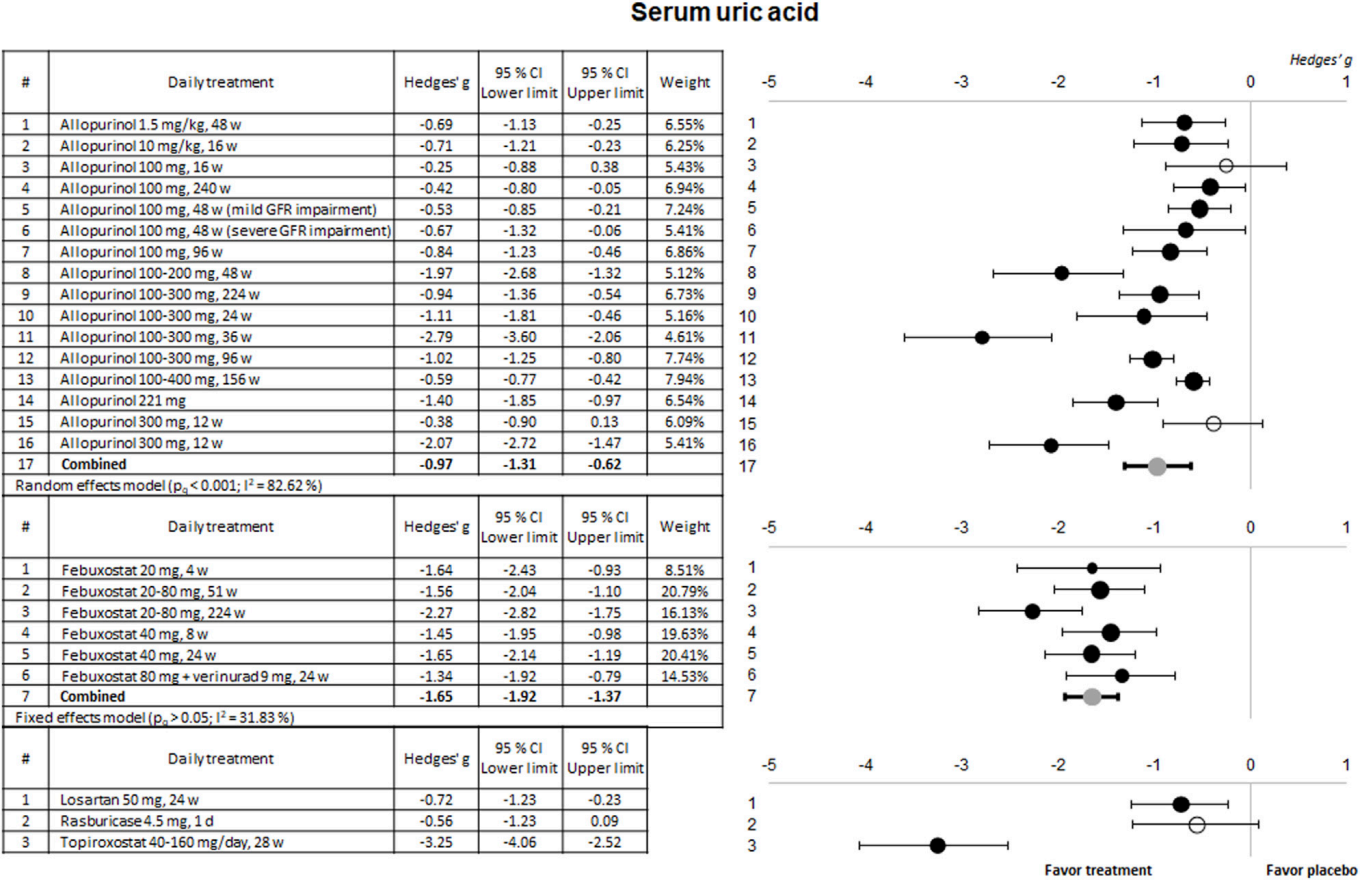
Meta-analytical results of the uric acid-lowering capacity of the therapies evaluated in the included clinical trials. Data are shown as a forest plot representing the difference in the means between the treated group and the control/placebo group for each trial. Effect size is measured as Hedges’ g ± 95% CI. CI: confidence interval; GFR, glomerular filtration rate; w, weeks.

The ability of the treatments to reduce sUA levels in the clinical trials included in this study is summarized in Figure 2. The two most evaluated compounds (allopurinol and febuxostat) significantly reduce sUA levels in 88% and 100% of the trials, respectively. Although higher for febuxostat, the combined meta-analytical result is very favorable for both drugs. In the case of allopurinol, higher effectiveness was observed for doses over 100 mg/day (regardless of the duration of treatment). In contrast, in the case of febuxostat, all tested doses (between 20 and 80 mg) showed similar beneficial effects. On the other hand, among the therapies evaluated to a lesser extent, only topiroxostat showed a highly significant hypouricemic effect.

#### 3.2.2 Nephroprotective effect

**FIGURE 3:**
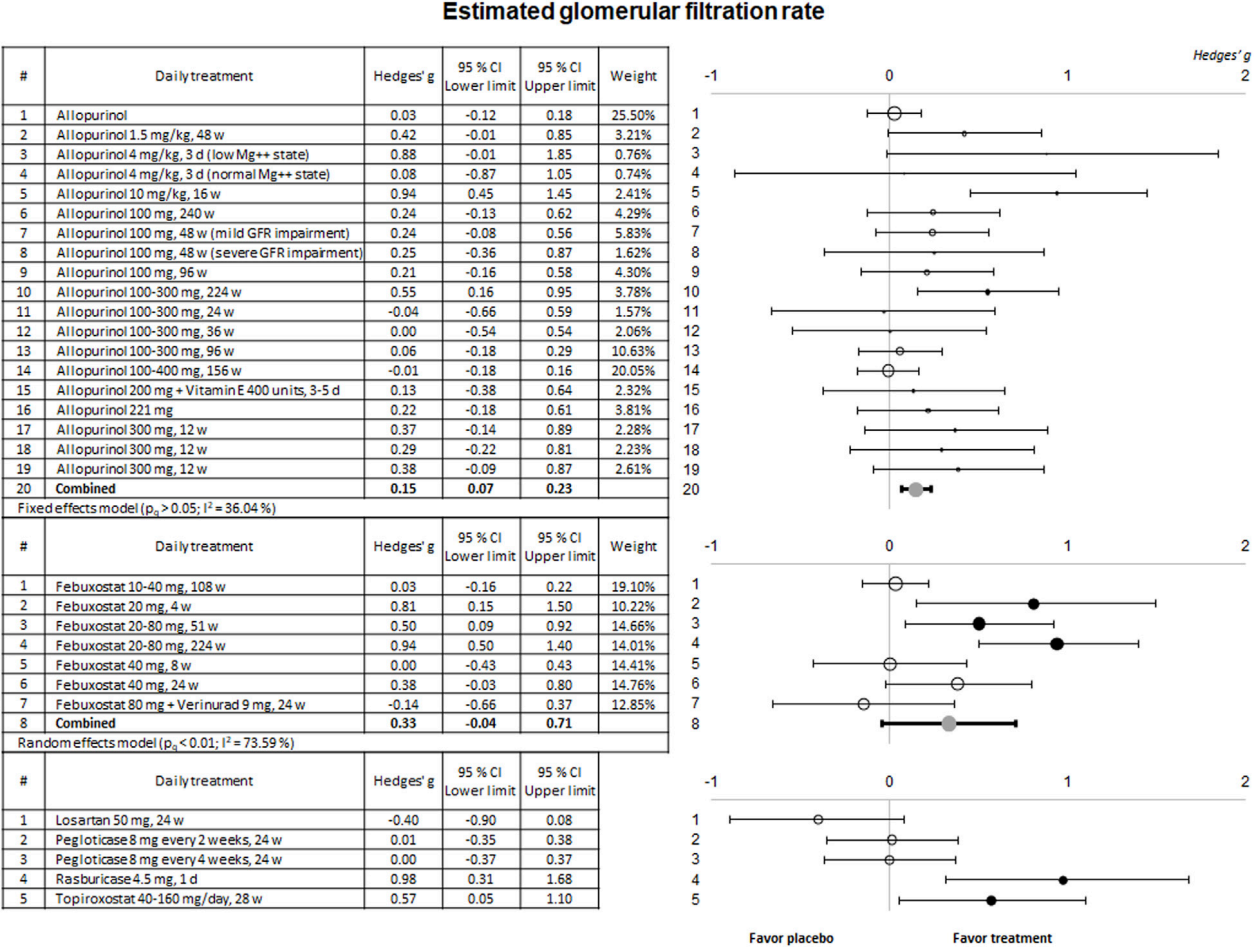
Meta-analytic results of the ability of evaluated therapies to improve or prevent eGFR deterioration. Data are shown as a forest plot representing the difference in the means between the treated and the control/placebo groups for each trial. Effect size is measured as Hedges’ g ± 95% CI. CI: confidence interval; d, days; eGFR, estimated glomerular filtration rate; w, weeks.

Most of the included trials evaluated the nephroprotective effect through the eGFR. The results of their meta-analysis are presented in Figure 3.

The combined result for allopurinol shows a significant nephroprotective effect. Of note, in practically all the studies, the effect on eGFR showed a very high interindividual variability. However, the tendency in almost all the studies using allopurinol shows a beneficial effect on their patients. In no case renal function worsened. In the case of febuxostat, only three studies using the 20 or 20–80 mg/day dosage demonstrated a significant nephroprotective effect. A beneficial effect on renal function was also seen in the only trial using rasburicase or topiroxostat.

**FIGURE 4:**
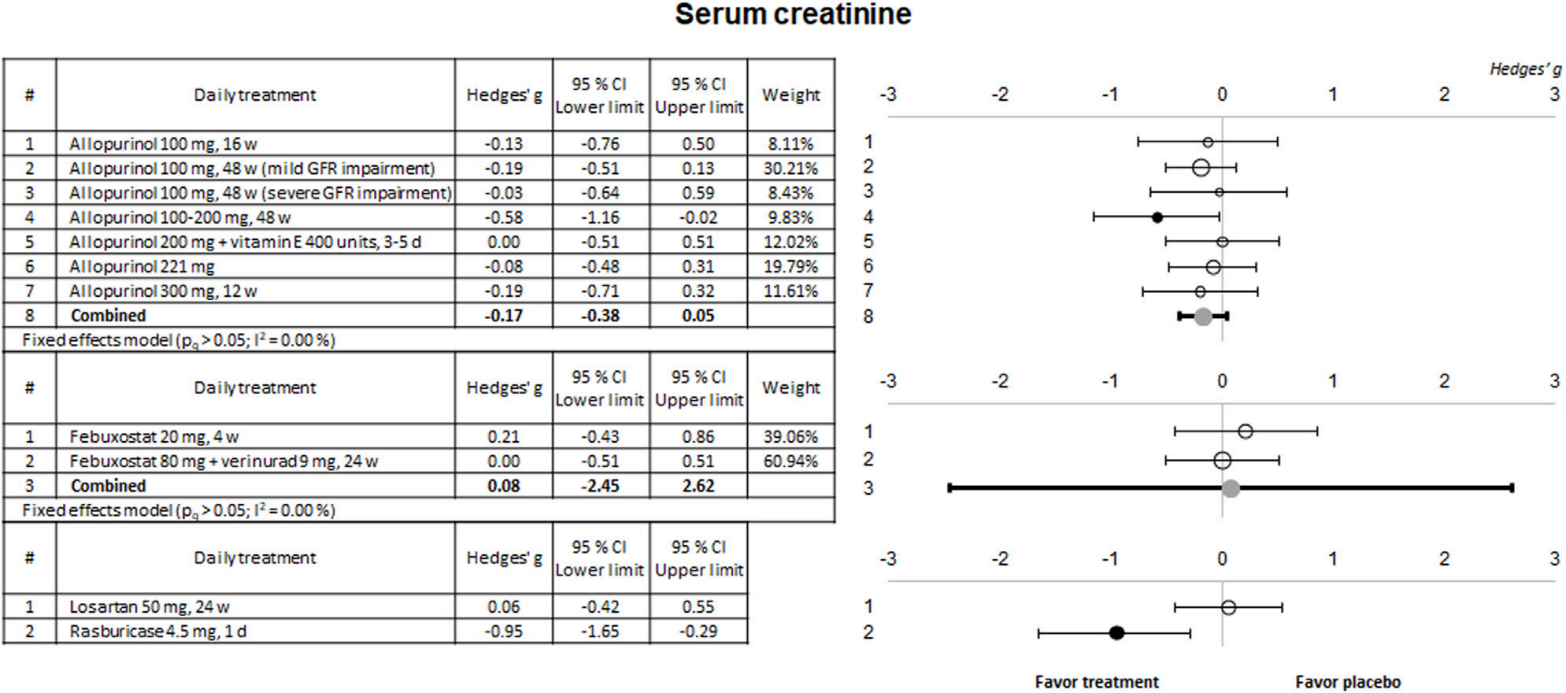
Meta-analytic results of the ability of evaluated therapies to reduce or prevent the in-crease of SCr levels. Data are shown as a forest plot showing the difference in the means between the treated and the control/placebo groups. Effect size is measured as Hedges’ g ± 95% CI. CI: confidence interval; d, days; GFR, glomerular filtration rate; w, weeks.

**FIGURE 5:**
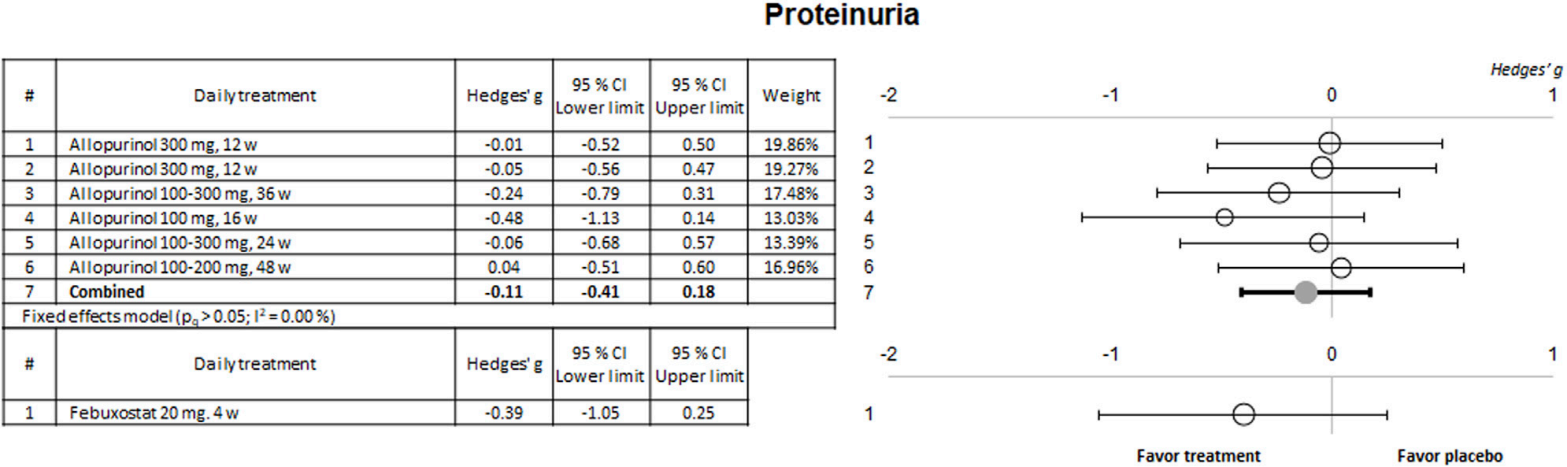
Meta-analytic results of the ability of evaluated therapies to reduce or prevent the in-crease in proteinuria. Data are shown as a forest plot showing the difference in the means between the treated and the control/placebo groups. Effect size is measured as Hedges’ g ± 95% CI. CI: confidence interval; w, weeks.

Some of the selected clinical trials also evaluated kidney function using sCr and urinary protein excretion. The results of the meta-analysis for these parameters are shown in Figures 4, 5, respectively.

No significant effects were observed on these two biomarkers for any of the drugs evaluated except for a slight nephroprotective effect observed in a study using 100–200 mg alopurinol, and an evident effect for rasburicase, both on sCr. No study showed a significant effect on proteinuria.

#### 3.2.3 Evaluation of publication bias

**FIGURE 6:**
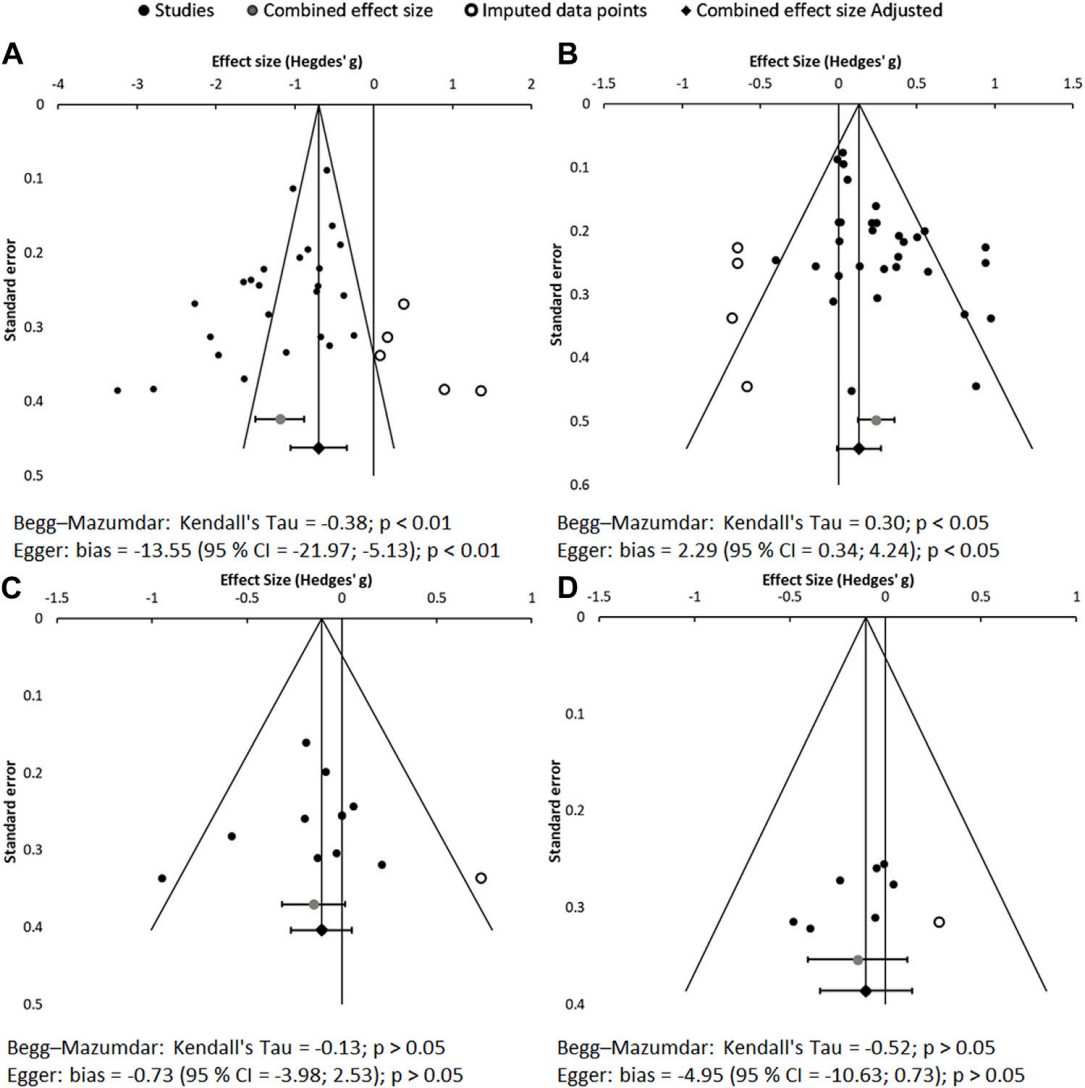
Funnel plots and asymmetry tests corresponding to the meta-analysis of sUA **(A)**, eGFR **(B)**, SCr **(C)** and proteinuria **(D)**. Effect size is measured as Hedges’ g ± 95% CI. CES: combined effect size; CI: confidence interval.

The results of the publication bias assessment are shown in Figure 6. Data distribution and asymmetry tests show a notorious publication bias (*p* < 0.01) for sUA. However, this bias does not affect the object of this meta-analysis, which is focused on whether the anti-gout, hypouricemic therapy exerts beneficial effects on CKD, not on whether the anti-gout therapy reduces uricemia. The asymmetry on sUA is expected, as all the drugs used in the included studies are known to be efficient at reducing hyperuricemia. Moreover, mild publication bias is also found for the eGFR. Specifically, graphical analysis identifies absence of treatments considerably worsening renal function. However, this is not real bias either, because all drugs tested are under clinical use. If deemed nephrotoxic, for ethical reasons they would never be administered to patients with CKD.

### 3.3 Correlation study

**FIGURE 7:**
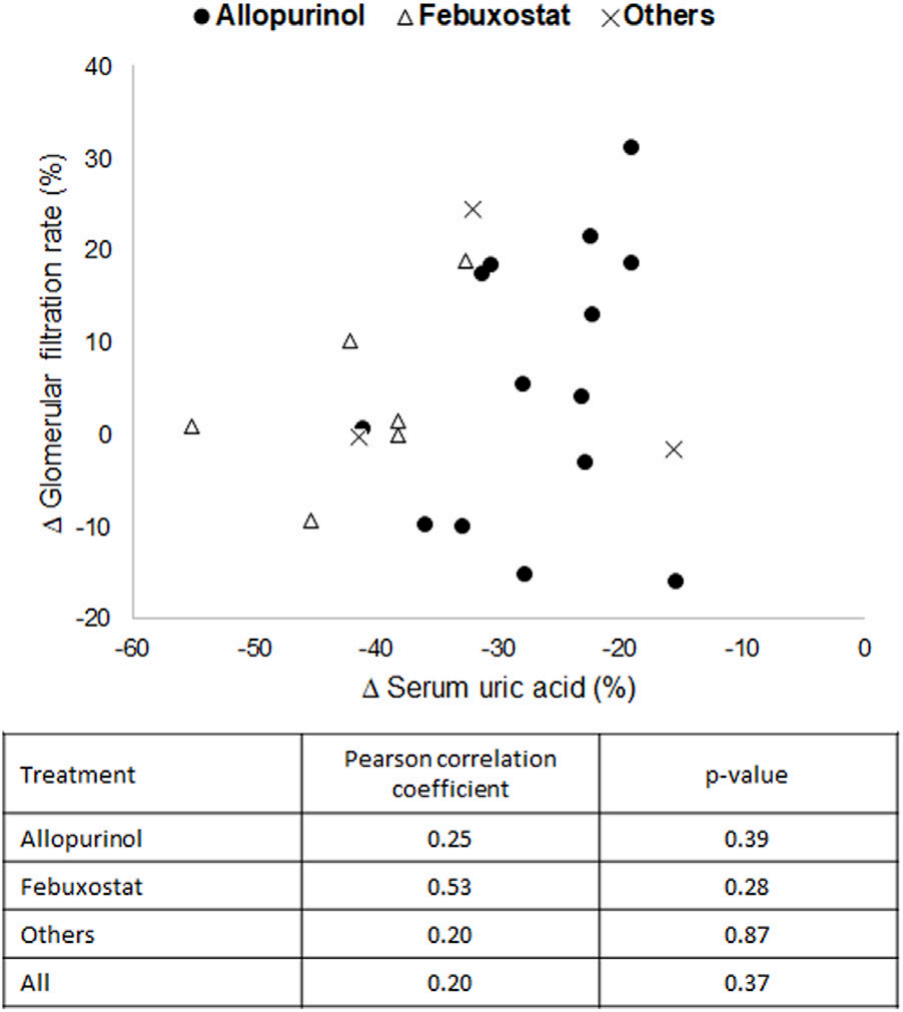
Graphical representation and correlation analysis of the average reductions in serum uric acid *versus* the average increases in estimated glomerular filtration rate.

The relationship between sUA reduction and improvement or prevent of eGFR deterioration (a standard renal function biomarker mostly evaluated in the included clinical trials) was studied with the Pearson test. As shown in Figure 7, no correlation was observed between both parameters for any of the drugs evaluated, nor for all of them in general. These results indicate that there is no direct or proportional relationship between them, which suggests that additional mechanisms other than the reduction of sUA contribute to the nephroprotective effect.

## 4 Discussion

Inclusion of a control/placebo group in the evaluation of the nephroprotective effect of hypouricemic therapy in CKD yields only a relevant number of studies for two drugs, allopurinol and febuxostat, from which convincing conclusions can be drawn. Our study demonstrates that both drugs positively affect glomerular filtration, with allopurinol showing a bolder effect. However, this limited casuistry is insufficient to infer whether there is a class effect of hypouricemic therapies on CKD, or the benefits exerted by allopurinol and febuxostat are due to additional mechanisms unrelated to the reduction of UA and specific of these two drugs. To overcome this limitation, a correlation analysis was carried out between the degree of UA reduction and the degree of renal protection for all the drugs included in the study. The results showed that reducing hyperuricemia is beneficial for renal function, but not in a directly proportional manner.

Proportionality might be disrupted by two factors. On the one hand, study population heterogeneity. A large inter-individual variability in the renal effect of antigout therapy is evidenced by the long error bars observed in virtually all the studies. This individual variability may be explained by the enrolment of patients at different stages of CKD and with different risk factors, whose renal damage patterns are heterogeneously caused by hyperuricemia and, thus, respond differentially to hypouricemic therapy. Identifying the phenotype and pathological scenario in which hypouricemic therapy provides nephroprotection to CKD patients poses an immediate research challenge. In this sense, some studies suggest that reducing UA may be more effective in preventing kidney damage in younger people (Feig, 2020) and in the early stages of CKD, which requires confirmation.

On the other hand, additional mechanisms unrelated to UA reduction could uncouple the apparent relationship of the hypouricemic effect on the nephroprotection observed between drugs and between patients. In this regard, allopurinol and febuxostat have strong antioxidant properties as both drugs are xanthine oxidase inhibitors (Becker et al., 2005; Schumacher et al., 2008). Several authors propose this mechanism as the main responsible for their nephroprotection (Okafor et al., 2017; Cicero et al., 2021). However, antioxidants alone are not enough to prevent CKD (Casanova et al., 2021). A marked anti-inflammatory effect on the vascular endothelium has also been reported for both allopurinol (Goicoechea et al., 2010) and febuxostat (Becker et al., 2005; Schumacher et al., 2008). Febuxostat has also been shown to prevent CKD progression in nephrectomized, normouricemic rats, by preserving preglomerular vessel morphology and maintaining glomerular pressure, which unveils an additional protective effect independently of UA levels (Sanchez-Lozada et al., 2018).

Another important observation from this meta-analysis is that, overall, renal protection is not dependent on drug dose or treatment duration. One possible reason is that in some clinical trials the dose was adjusted as treatment progressed, while in others it was not. This could affect the results since possibly not all patients received the most appropriate dose.

Interestingly, protection has been observed even in short-term treatments (i.e., 4–16 weeks) (Kanbay et al., 2007; Momeni et al., 2010; Bayram et al., 2015; Ghane Sharbaf and Assadi, 2018; Perrenoud et al., 2020; Wen et al., 2020). This suggests that, in addition to impinging on slower chronic processes of kidney injury underlying CKD progression, hypouricemic agents may also ameliorate renal function by a relatively swift mechanism. For instance, an improvement in endothelial function bestowed by antioxidant and anti-inflammatory effects and by increased nitric oxide availability (Khosla et al., 2005; Schwartz et al., 2011), may cause renal vasodilation, increase renal blood flow and GFR, and explain the faster response seen in some patients. Distinct acute effects also contribute to the variable effect exerted by different drugs, and for the same drug between patients.

Thus, the overall effect of a drug in a specific study depends on the heterogeneous composition of pathophysiological patterns in the population studied, as determined stochastically or by environmental and social factors. Individual responses depend, in turn, on how the pharmacological mechanisms of the drug used match the patient’s pathophysiological pattern. In perspective, personalized antigout therapy in CKD should be based on the election of the appropriate drug/dose for each scenario.

Of note, only two of the 29 articles included in this meta-analysis considered albuminuria in assessing renal function, despite its importance in the diagnosis of CKD (Hallan et al., 2009). In fact, albuminuria complements the GFR, improves CKD diagnosis, risk stratification, and prognosis of progression (Hallan et al., 2009; Polkinghorne, 2014; Lambers Heerspink and Gansevoort, 2015), and forms part of the Kidney Disease Improving Global Outcomes guidelines (i.e., the international consensus diagnostic criteria) since 2012 (KDIGO, 2013). While GFR only informs on status of the glomerular filtration process, increased albuminuria may reflect a change in glomerular permselectivity and a defect in tubular function, specifically in tubular reabsorption (Levey et al., 2020; Divya et al., 2023). Incomplete renal function diagnosis may cause misinterpretation of the effect of hypouricemic therapies. Curiously, this affects studies performed both before and after public availability of the KDIGO guidelines. We contend that future clinical trials designed to evaluate the nephroprotective effect of UA-lowering therapy should collect both GFR and albuminuria values for a more granular detection of the pathophysiological spectrum underlying CKD.

In conclusion, this work shows that the reduction of hyperuricemia might potentially be an effective strategy in the prevention of CKD in specific pathological scenarios, which needs to be further explored. The phenotype of patients who may benefit from this therapy should be the focus of future studies.

## Data Availability

The raw data supporting the conclusion of this article will be made available by the authors, without undue reservation.
